# Access to child developmental assessment services in culturally and linguistically diverse metropolitan Sydney: a retrospective cohort analysis

**DOI:** 10.1186/s12913-024-10800-y

**Published:** 2024-03-14

**Authors:** Sibella E. Bentley, Pankaj Garg, Ori Gudes, Romy Hurwitz, Sinthu Vivekanandarajah, Lydia Y.L. So

**Affiliations:** 1 The Children’s Hospital at Westmead, Corner Hawkesbury Road and Hainsworth Street, Westmead, NSW 2145 Australia; 2 Department of Community Paediatrics, South Western Sydney Local Health District Health Services Building Level 3, Liverpool, NSW 2170 Australia; 3 Department of Medicine & Health, University of New South Wales, Sydney, Australia; 4 School of Population Health, University of New South Wales, Sydney, Australia; 5 University of Notre Dame Australia School of Medicine, Sydney, Australia

**Keywords:** Developmental assessment, Cultural and linguistic diversity, Wait times, Access, Vulnerability

## Abstract

**Background:**

Despite the increasing prevalence of neurodevelopmental disorders (NDD), data regarding access to child development services have remained limited globally. Long wait times are a major barrier to developmental assessments, impacting on care and outcomes. The aim is to retrospectively analyse the demographic profile and prioritisation of patients seen at a child developmental assessment service (CDAS) in a vulnerable region of Sydney, and explore factors affecting wait times.

**Methods:**

Data was collated and analysed for 2354 patients from 2018 to 2022. Socio-Economic Indexes for Areas (SEIFA) were collated from the Australian Bureau of Statistics. Descriptive statistics were used for demographic data and various statistical methods were used to analyse the relationships and impact of factors likely to affect wait lists.

**Results:**

The median age was 51 months (IQR41-61) and males comprised 73.7% of the cohort. 64% of children were from culturally and linguistically diverse backgrounds (CALD) and 47% lived in the most disadvantaged suburbs. The median wait time was 302.5 days (IQR175-379) and 70% of children were seen within 12 months. CALD patients and children over 5-years had shorter wait times. Most children with Global Developmental Delay (GDD) were from the lowest four SEIFA deciles and waited longer for an appointment. 42.6% were seen within the priority allocated time or sooner. Children with ASD and/or severe GDD were prioritised to be seen earlier. Overall, the study could not demonstrate any difference in the wait times according to the prioritisation groups.

**Conclusion:**

This study provides insights into the profile, prioritisation processes and wait lists of children seen by CDAS in South Western Sydney with high rates of social vulnerability and presents an argument to discuss benchmarking targets with service providers. It identifies the need to prioritise children living in suburbs with socioeconomic disadvantage and refine prioritisation and data collection processes to improve wait times.

**Supplementary Information:**

The online version contains supplementary material available at 10.1186/s12913-024-10800-y.

## Background

There is an increasing prevalence of neurodevelopmental disorders (NDD) and a growing need for early identification, assessment and intervention [1–4]. A significant proportion of outpatient work for primary care paediatricians involves identification and management of NDD [5, 6]. However, many children require a more comprehensive multidisciplinary developmental assessment due to severity, complexity or diagnostic uncertainty for the purposes of accessing appropriate educational and interventional supports [7]. Furthermore, when clinicians are unsupported by the health systems and funding mechanisms, they often refer children for tertiary assessments [3, 8]. Parents also report better understanding of their child’s needs when they receive a multidisciplinary assessment [9]. Evidence indicates that multidisciplinary child diagnostic services are the best practice for evaluating children with complex NDD [10].

A major barrier to accessing publicly funded paediatric developmental clinics are lengthy wait times [11–13]. There is a significant gap in the literature on this matter [2]. It is relevant, given the time-sensitive nature of early intervention, to maximise developmental gains and reduce functional limitations [10, 11, 14]. Studies in North America found that families waited over two years for a diagnosis and in British Columbia, children waited 12–18 months for an ASD diagnostic assessment [2, 12]. Long waiting lists can also deter clinicians from referring to these services [3, 12, 13]. One study identified that 30% of children referred for assessment of ASD were not ultimately diagnosed with ASD [15]. Therefore, referrals to child development services can be for diagnostic clarification only and has implications for training of paediatricians.

Children with NDD are vulnerable members of the community and often come from socially disadvantaged families [10, 16]. Research has shown that social isolation, psychosocial risk and poor health literacy can delay access to developmental services [2, 17]. Vulnerable populations, therefore, enter developmental diagnostic services with lower self-efficacy scores compared to non-vulnerable populations [18]. It is known that children from culturally and linguistically diverse (CALD) backgrounds can start school with undiagnosed NDD and are less likely to receive services [17]. In Australia, children with an intellectual disability are more likely to be exposed to a lower socioeconomic environment [16]. Those with higher socioeconomic backgrounds have better outcomes and the quality of early intervention in disadvantaged areas is reported to be poorer than other areas [16].

The Child Development Assessment Service (CDAS) in South Western Sydney (SWS) Local Health District (LHD) provides developmental assessments, diagnostic formulation and management recommendations. CDAS is a multidisciplinary team of clinicians which is staffed by paediatricians, paediatric trainees, psychologists, occupational therapists, speech pathologists and social workers depending on the assessment needs of each child. CDAS covers one of the most disadvantaged regions with regards to education, employment and income as measured using the Socio-Economic Indexes for Areas (SEIFA) from the Australian Bureau of Statistics (ABS) [19, 20]. There is a high population of CALD peoples in SWS whereby in 2016, 64% of households spoke a language other than English, and a greater proportion of people were born overseas compared to the rest of the state [20].

To meet the growing demand and streamline access to developmental services in SWSLHD, a redesign of CDAS was undertaken in 2016 and a comprehensive model of care was developed based on available research evidence and principles of best practice (CDAS Model of Care, supplementary file). This involved creating a standardised intake process, prioritisation and a central database to ensure consistent prospective data collection [21]. The intake and triage process included gathering of information on children who will require various levels of multidisciplinary clinic and who will be most suitable for paediatrician/allied health only clinic. The service developed two separate pathways for preschool, and school aged children as clinicians intended to prioritise children before their first year of formal schooling. In the absence of evidence-based guidance, the timelines for priority allocation were based on the consensus from a group of developmental paediatricians. The team developed priority allocations of 1, 2, and 3 to be seen within 3, 6 and 12 months respectively based on clinicians’ judgement of child, referral and family factors. For example, CDAS prioritises children who had no assessments, access to paediatricians, or clear diagnosis, or other complex neurodevelopmental concerns and were reaching the age of first year of formal schooling. Prioritisation for multidisciplinary teams was also based on which children were likely to have significant symptoms of ASD and require diagnostic clarification. These priority allocations were fluid and could change if additional important child or family related information became available (CDAS Model of Care, supplementary file).

To date, no published research exists that has provided a comprehensive analysis of waiting times, prioritisation and access to publicly funded developmental assessments and how this relates to the markers of social vulnerability. This research gap challenges the ability of services to benchmark, target resources and systematically address the issue of long waiting times [2]. The allocation of additional resources to these services by health managers can be difficult in the absence of an adequate evidence-base that describes the children on wait lists. This analysis would provide data for making quality improvement recommendations on benchmarking standards.

## Method

### Data

SWSLHD CDAS dataset was extracted over a four-year period from August 2018 (when consistent data collection started) until September 2022. Demographic data included gender, suburb of residence, country of birth, priority allocated, Indigenous status, CALD, whether the child is known to the Department of Community and Justice Services (DCJ), in out-of-home care (OOHC), refugee status, date of referral, date seen, facility, primary diagnosis, level of delay and outcome of assessment. There are five community health centres within the service and these have been de-identified as Facilities A-E. The study looked for variability in the proportion of children seen in the different facilities. This was to identify areas that need more targeted distribution of resources and to refine prioritization processes. All centres had similar capacities of assessment and tools to perform standardised neurodevelopmental assessments. Staffing of paediatricians was variable and dependent on longer waiting lists. There was mobility of medical staff as all centres were under the umbrella of one unified department.

### SEIFA indices

To determine the suburban level measure of relative socioeconomic disadvantage, SEIFA indices from the ABS website were downloaded [19]. Local Government Areas (LGA) are given an Index of Relative Socio-Economic Disadvantage (IRSD). Deciles (from lowest 1 to highest 10) as well as IRSD scores [mean (SD): 1000(100)], were extracted and utilised for analysis [19]. A lower score indicates that an area is relatively more disadvantaged compared to an area with a higher score.

### Data cleaning

**Fig. 1:**
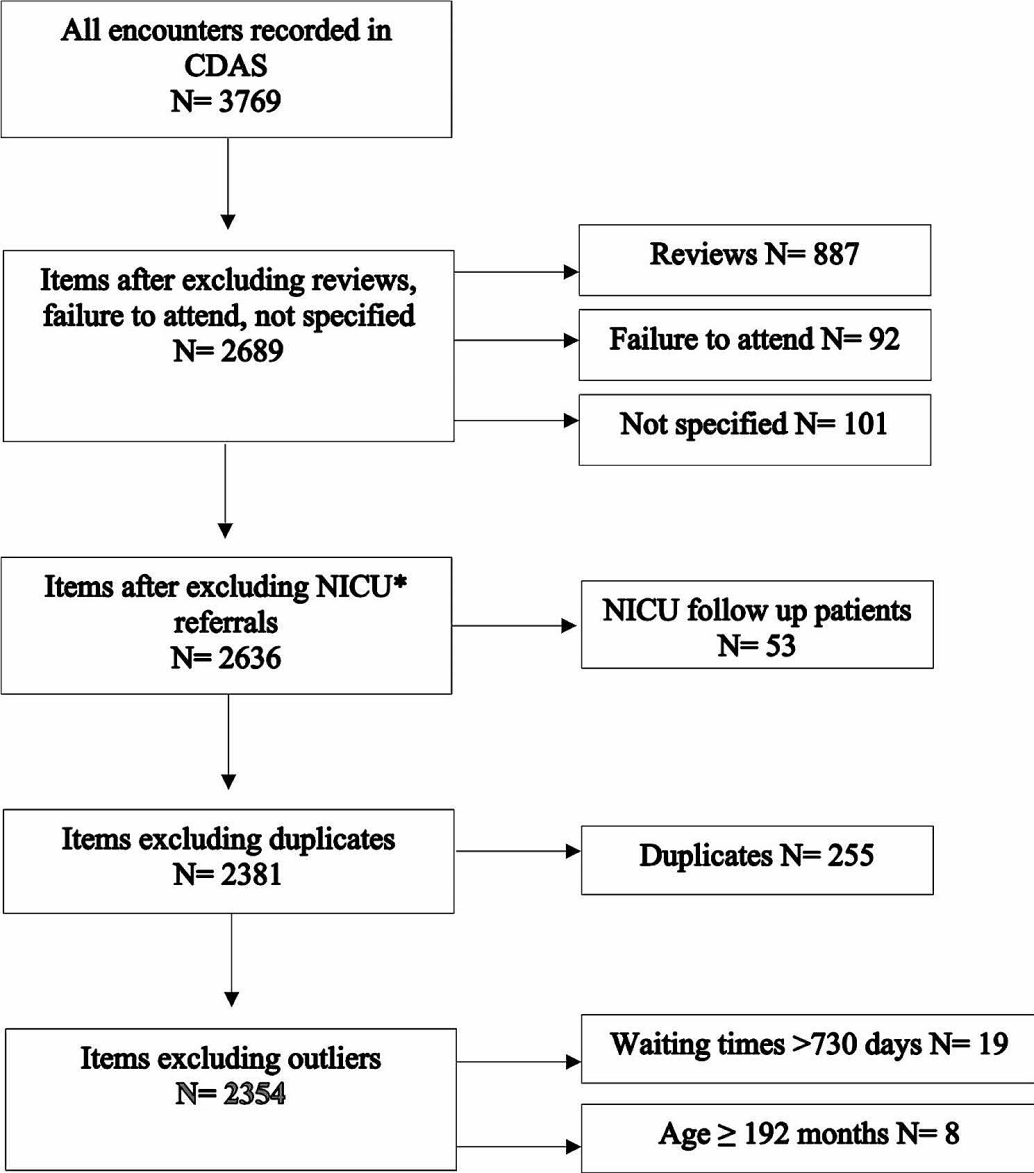
Flow diagram of included patients seen in CDAS clinic during the first encounter. *Neonatal Intensive Care Unit (NICU) – included patients that were entered from neonatal follow up program, and were not CDAS patients

A data cleaning process was performed (Fig. 1**).** Initial data consisted of 3769 encounters. As the purpose of the study was to look at the initial visit only, all reviews and failure to attend records were deleted. Outliers were also removed, that included patients who waited more than 730 days (2-years) and children who were 192 months (16-years) or older at the time of visit. These children were excluded as patients older than 16-years are not typically seen by CDAS. Those waiting more than two years were an exceptional group due to complex family level factors such as interstate travel and required a separate qualitative data analysis. These outliers formed less than 1% of the total dataset.

### Data analysis

Descriptive statistics was used to report on the demographic data. To address the impact of factors on wait times, one-way Analysis of Variance (ANOVA) was used. We have presented the F-ratios to highlight the source of variations in the groups in ANOVA analysis. F-ratio is the ratio of mean square between group fluctuations and within group fluctuations. Large F-ratios reflect large variability in the data. Where the relationship between two continuous variables was curvilinear in nature that was evident from the visualization of data from ANOVA analysis, a polynomial regression analysis was performed [22]. Models with lines of increasing degree of order were used to find the best fit for the data scatter. Under and over-fitting was avoided by visualizing the line of model fit. As the relationship between some continuous variables was complex, R- squared was not considered a good measure for model of fit. Instead, a standard error of regression indicated how far the data points were from the regression line on average [22]. Chi-squared analysis was performed to examine bivariate analysis of correlation between priority allocated and other factors. MedCalc for Windows, version 19.4 (MedCalc Software, Ostend, Belgium) and Windows Excel, version 16.16.27 were used for statistical analysis. Python code was used for Polynomial regression [23].

### Ethics approval

Institutional approval was granted by the district Quality Improvement Committee (ID 2745). Consent was waived as there was no patient contact and all data was de-identified.

## Results

### Demographics

Data was analysed from 2354 patients. Table 1 summarises the demographic data for the study cohort. The median age of new patients seen was 51 months (IQR 41–61). Most of our cohort were children less than seven years (2142/2354, 91%). The majority of patients were boys (73.7%) and were between 3 and 5 years of age (61.1%). In the cohort, 64% were from CALD backgrounds. The majority of children were born in Australia (89.6%). The highest ethnicity group was Asian (32.7%), followed by white ancestries (19.8%) and Arabic background (15.9%). One-third (34%) received a priority 1 triage and 26% received priority 3. The number of children seen fluctuated with highest numbers seen in 2019 (*n* = 679). Facility C (40.8%) saw the greatest number of children whilst B (3.7%) saw the least. The highest percentage of patients lived in decile 1 and 2 suburbs (47%) and the least proportion of children resided in deciles 7 and 8 suburbs (9.1%). Approximately one-quarter (27.5%) of children had mild delays, whereas 38% of children had moderate to severe delays. For 8.7% of children, the level of delay was considered significant but not classified further. The most common reported diagnosis was GDD either alone or associated with ASD (62.5%), followed by ASD (53%) (Table 1). There was variability in the proportion of children residing in different levels of disadvantage. Table 2 demonstrates a percentage breakdown comparing suburb of residence IRSD deciles between facilities. Facility D had the highest proportion of patients living in suburbs classified in the lowest two deciles (90.9%).

**Table 1:** Demographic data of patients seen on initial assessment visit with CDAS

Characteristic	n (%)
**Gender**	
Male Female	1732 (73.7) 618 (26.3)
**Age at presentation** (months)*	51 (41-61)
**Age group**	
<3 years 3-5 years >5 years	311 (13.2) 1438 (61.1) 605 (25.7)
**Country of birth**	
Australia Outside Australia	2097 (89.6) 257 (10.4)
**Indigenous Status**	
Indigenous status Non-Indigenous status	130 (5.9) 2086 (94.1)
**Culturally and linguistically diverse**	
Yes No	1389 (64.0) 782 (36.0)
**Ethnicity**	
Arabic Asian African Hispanic Mixed Pacific Islander Caucasian Unknown	309 (15.9) 636 (32.7) 57 (2.9) 21 (1.1) 190 (9.8) 50 (2.6) 584 (19.8) 408 (21.0)
**In out-of-home care**	52 (2.2)
**Known to Department of Communities and Justice**	64 (2.7)
**Refugee or Asylum**	40 (1.7)
**Year seen**	
2018 (from August) 2019 2020 2021 2022 (until September)	177 (7.5) 679 (28.8) 577 (24.5) 592 (25.1) 29 (14.0)
**Facility seen**	
A B C D E	265 (11.3) 87 (3.7) 960 (40.8) 328 (13.9) 714 (30.3)
**Priority allocated data**	
Priority 1 Priority 2 Priority 3	768 (34.4) 890 (39.8) 577 (25.8)
**Index of relative socio-economic disadvantage**	
Decile 1-2 Decile 3-4 Decile 5-6 Decile 7-8 Decile 9-10	1107 (47.0) 371 (15.8) 270 (11.5) 215 (9.1) 319 (13.6)
**Diagnosis**	
Autism Spectrum Disorder Global Developmental Delay Autism Spectrum Disorder and Global Developmental Delay Intellectual Disability Specific Language Impairment Average Not available	462 (23.6) 655 (33.1) 581 (29.4) 15 (0.8) 65 (3.3) 113 (5.7) 87 (4.4)
**Level of Delay**	
Mild Moderate Severe Profound Significant Above average and average Borderline and low average	537 (27.5) 537 (27.5) 214 (11.0) 15 (0.77) 170 (8.7) 273 (14.0) 208 (10.6)

*N*= 2354. *Median (25-75 IQR); **Patients receive 1 point for each vulnerability factor of Indigenous, OOHC, refugee and known to DCJ. Categories of average, above average, borderline and low average were considered not be developmentally delayed compared to others

**Table 2:** Comparing patient suburb of residence IRSD decile between facilities

IRSD Decile	Facility n (%)				
	A n(%)	B n(%)	C n(%)	D n(%)	E n(%)
**1–2**	175 (66.0)	6 (6.9)	251 (26.1)	298 (90.9)	377 (52.8)
**3–4**	18 (6.8)	16 (18.4)	222 (23.1)	11 (3.4)	104 (14.6)
**5–6**	39 (14.7)	35 (40.2)	163 (17.0)	7 (2.1)	26 (3.6)
**7–8**	13 (4.9)	17 (19.5)	63 (6.6)	6 (1.8)	116 (16.2)
**9–10**	20 (7.5)	13 (14.9)	261 (27.2)	6 (1.8)	91 (12.7)

IRSD- Index of relative social-economic disadvantage

### Waiting times

The overall median waiting time was 302.5 days (IQR175-379) [9.9 months (IQR 5.8–12.5)], and mean was 291 days (95% CI, 284.7 to 297.6) [9.6 months (9.3–9.8)]. About one-tenth of children were seen within 90 days, about 25% children within six months and 70% within 12 months. Almost one-third (30.9%) waited for more than 12 months (Table 3). There were 42.6% of children who were seen within the priority allocated at the time of triage or earlier than the allocated priority.

**Fig. 2:**
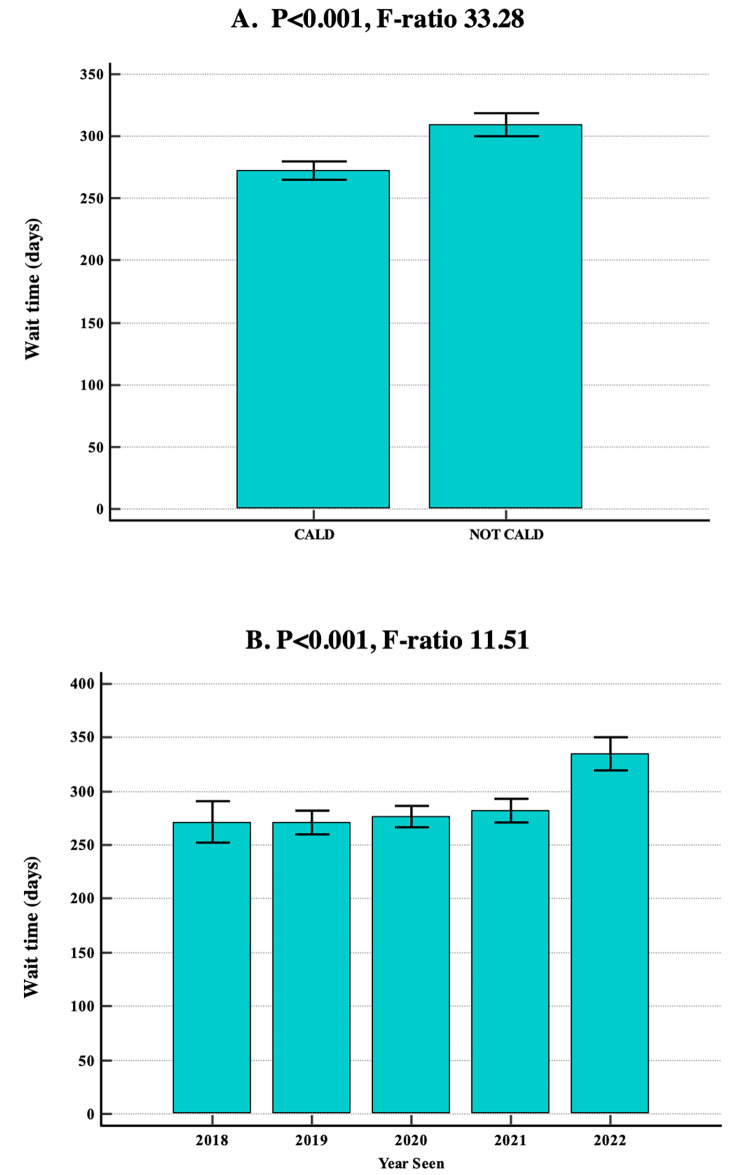
Impact of facility, CALD and year seen on wait time in days. Utilised ANOVA with 95% confidence intervals. A: Comparison of mean wait times between CALD and non-CALD patients (*p* < 0.001). B: Comparison of mean wait times between the year patients were seen (*p* < 0.001). F-ratios are presented to highlight the variability between and within groups

**Fig. 3:**
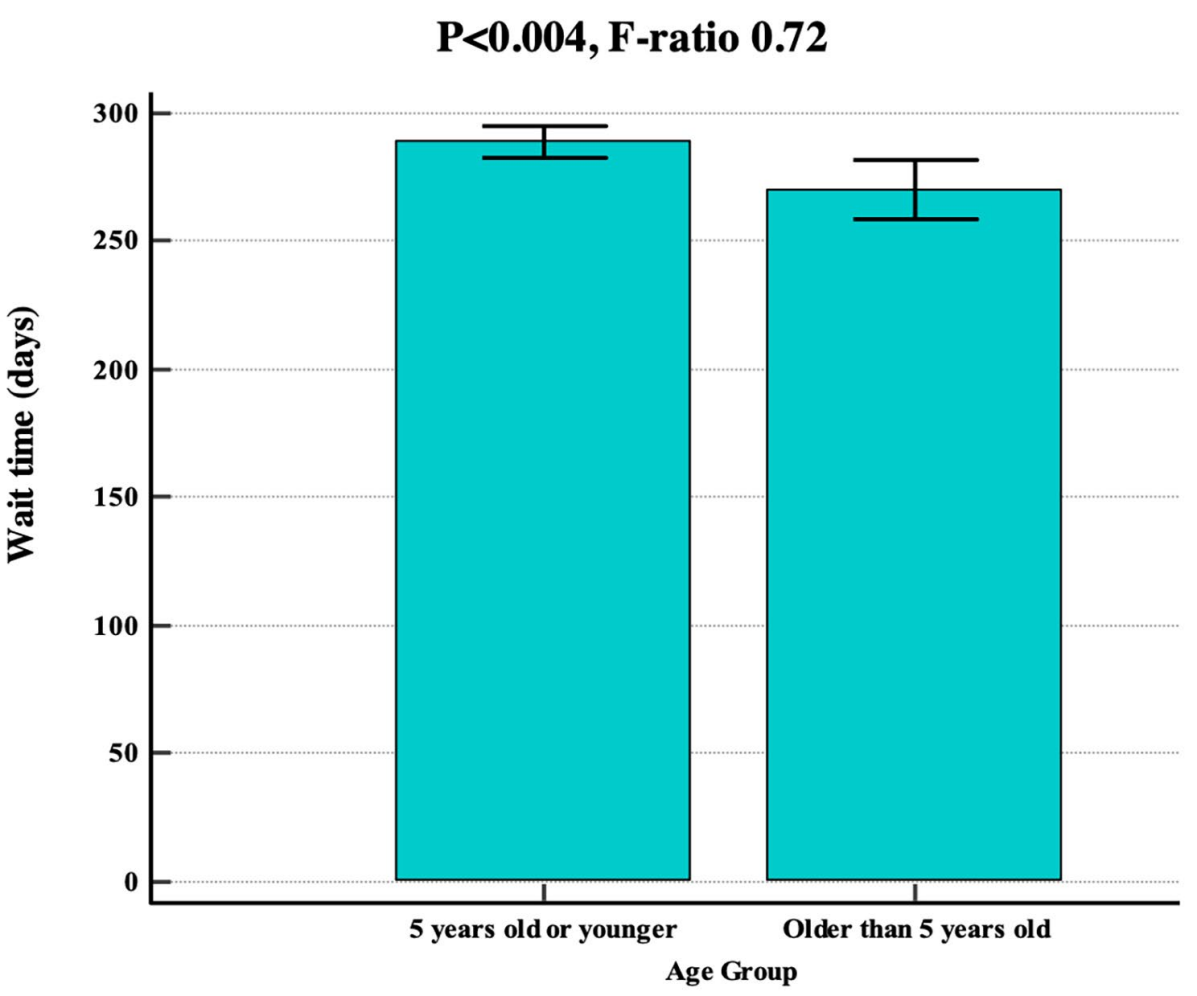
Impact of age group on wait times in days. Utilised ANOVA with 95% confidence intervals and polynomial regression. F-ratios are presented to highlight the variability between groups

There were significant differences in the wait times according to the facility with C having longer wait times than A, B, D and E (*p* < 0.001; 95% CI 337.8-353.2). Patients from CALD backgrounds had a statistically significant shorter mean wait time of 272.2 days (SD = 137) compared to those from non-CALD backgrounds (mean = 309.3 days, SD = 132) (*p* < 0.001; 95% CI 265.1-279.3, Fig. 2A). Wait times in 2022 (mean = 334.8 days, SD = 144) were longer compared to previous years (*p* < 0.001; 95% CI 320.3-349.3, Fig. 2B). Children older than 5-years were seen sooner (mean = 270.3 days, SD = 146) compared to children 5-years and younger (mean = 288.9 days, SD = 132) (*p* < 0.004; 95% CI 259.4-281.1, Fig. 3). There was no significant difference in mean wait times between gender (*p* < 0.599).

**Fig. 4:**
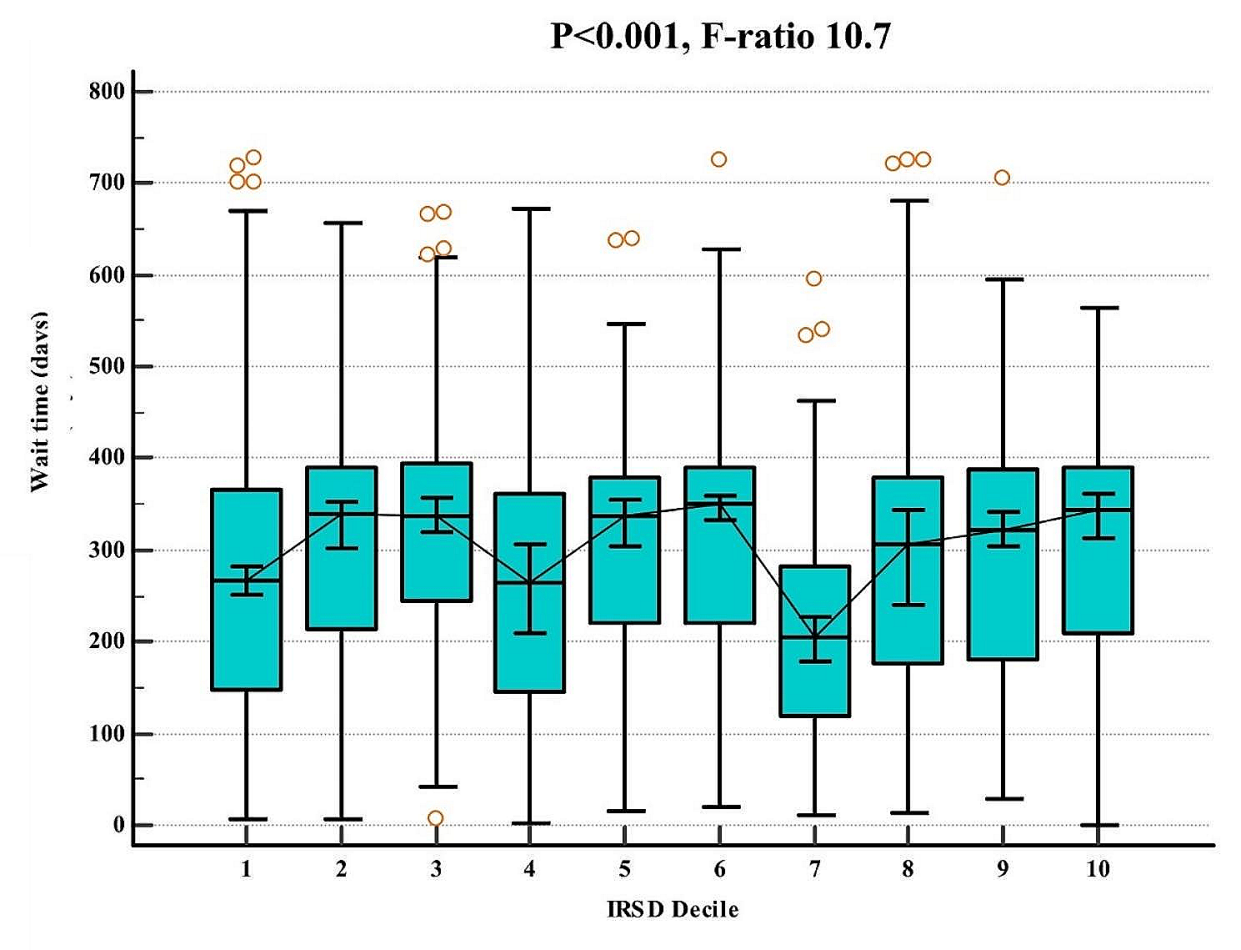
Comparison of mean wait times with IRSD deciles with 95% confidence intervals. IRSD- index of relative socio-economic disadvantage. F-ratio is presented to highlight the variability between and within groups

There was variability in the wait times according to IRSD deciles. IRSD deciles 7–8 had a shorter wait time (mean = 147.4 days, SD = 147) compared to deciles 3–4, 5–6 and 9–10 (95% CI 235-271.5 vs. 95% CI 281.4-292.9). Deciles 1–2 had a shorter mean wait time of 273.8 days (SD = 274) compared to deciles 5–6 (314.4 days) (*p* < 0.001, 95% CI 265-281.8 vs. 95% CI 298.2-330.5). Figure 4 highlights the variability of wait times with each IRSD decile at a population level.

Children seen through CDAS with mild, moderate, severe and profound GDD lived in suburbs rated in the lowest four deciles for socio-economic disadvantage. All children with profound delays lived in the most disadvantaged suburbs.

**Table 3:** Median wait times according to demographics and vulnerability

Variable (days)	Median (IQR 25–75)
**Overall wait times**	302.5 (175–379)
Wait time groups*	
<=90 days 91–180 days 181–365 days > 365 days	225 (9.6%) 390 (16.6%) 1011 (42.9%) 728 (30.9%)
**Wait times according to facility**	
A B C D E	282 (167-357.8) 274 (215–265) 365 (306–399) 308 (196-386.5) 179 (112–279)
**Wait times according to year seen**	
2018 2019 2020 2021 2022	308 (159–372) 288 (145–384) 300 (183-358.3) 282 (186–382) 363 (269-420.3)
**Wait times according to age group**	
< 3 years 3–5 years > 5 years	298 (175.5–372) 309.5 (194–381) 280 (147-375.3)
**Wait times according to IRSD**	
Decile 1–2 Decile 3–4 Decile 5–6 Decile 7–8 Decile 9–10	284 (161.3–374) 322 (195-381.8) 344 (221–385) 233 (141.5-344.8) 333 (189.3–389)
**Wait times according to priority**	
1 2 3	272 (147–282) 256 (180–275) 264 (285–385)

*Frequency N (percentage %). IRSD- index of relative socio-economic disadvantage

### Multidisciplinary group and priority allocation

Age groups of less than 3 years, 3–5 years and older than 5 years had a statistically significant difference in priority allocation (*p* < 0.0001, 95% CI 261.6-291.8, 284.5-298.5, 259.5-281.1 respectively) (Table 4). There was also a significant difference in priority allocation to CALD patients compared to non-CALD (*p* < 0.0001). Children with severe and profound GDD were prioritised more urgently in category 1 compared to category 2 (*p* = 0.008) (Table 4). Children with ASD and ASD/GDD were also prioritised more urgently (*p* < 0.0001; *p* = 0.004 respectively). Overall, there were no demonstrated differences in the wait times according to prioritisation groups. The data on multidisciplinary allocation was not collected with accuracy so was not analysed and presented.

**Table 4:** Chi-analysis of priority allocated between age groups, CALD background, outcome assessment, level of delay and primary diagnosis

Variable	Priority Allocated				DF	p	Chi-squared		
**Age Groups**	**1**	**2**	**3**		4	**< 0.0001**	41.328		
< 3 years	83(10.8%)	98(11.0%)	114(19.7%)	295 (13.2%)					
3–5 years	454(59.1%)	585(65.7%)	340 (59.0%)	1379 (61.7%)					
> 5 years	231(30.1)	207(23.3%)	123 (21.3)	561 (25.1%)					
Total (n)	768 (34.4%)	890 (39.8%)	577 (25.8%)	2235					
**CALD Status**	**1**	**2**	**3**		2	**< 0.0001**	25.529		
CALD	475(65.7%)	560(69.5%)	307(56.2%)	1342 (64.7%)					
Not CALD	248(34.3%)	246(30.5%)	239(43.8%)	733 (35.3%)					
Total (n)	723 (34.8%)	806 (38.8%)	546 (26.3%)	2075					
**Level of Delay**	**1**	**2**	**3**		6	**0.008**	17.529		
Mild	179(35.2%)	231(36.8%)	119(33.8%)	529 (35.6%)					
Moderate	160(31.5%)	229(36.5%)	133(37.8%)	522 (35.1%)					
Severe	85(16.7%)	71(11.3%)	55(15.6%)	211 (14.2%)					
Profound Significant Borderline	10(2.0%) 53(10.4%) 21(4.2%)	2(0.3%) 61(9.7%) 33(5.3%)	3 (0.9%) 34(9.7%) 8(2.2%)	15 (1.0%) 148 (9.9%) 62 (4.2%)					
Total (n)	508 (34.1%)	627(42.2%)	352 (23.7%)	1487					
**Primary Diagnosis**	**1**	**2**	**3**		12	**< 0.0001**		59.630	
ASD	204 (29.6%)	149(18.9%)	94(21.8%)	447 (23.4%)					
ASD/GDD	191(27.7%)	207(26.2%)	168(39.0%)	566 (29.6%)					
Average	41(6.0%)	53(6.7%)	18(4.2%)	112 (5.9%)					
GDD	207(30.0%)	301(38.1%)	124(28.8%)	632 (33.1%)					
ID	3(0.4%)	8(1.0%)	2(0.5%)	13 (0.7%)					
Not Applicable	26(3.8%)	35(4.4%)	17(3.9%)	78 (4.1%)					
SLI	17(2.5%)	36(4.7%)	8(1.8%)	61 (3.2%)					
Total (n)	689 (36.1%)	789 (41.3%)	431 (22.6%)	1909					

*P < 0.05 statistically significant. ID- intellectual disability; SLI- specific language impairment, average- normal range developmental skills

## Discussion

Our study is the first to provide a comprehensive analysis of waiting times, prioritisation and access to publicly funded developmental assessments and how this relates to the markers of social vulnerability. Our study is thus filling a major gap in the literature for access and waiting times. It will help further research on providing guidance on benchmarking, targeting resources and to systematically address the issue of long waiting times. Our cohort was representative of the population with people from diverse communities and speaking various languages residing in this region of metropolitan Sydney.

Our study analysis of 2354 patients demonstrated that the median wait time for an initial CDAS appointment was 302.5 days (IQR: 175–379). The service saw 25% of children within 6 months and 70% within 12 months. However, almost one-third waited over 12 months. There was no difference in median wait times between children seen in priority 1, 2 or 3 as more than half of the children could not be seen within the priority allocated at the time of triage affecting wait times in each group.

The clinicians prioritised CALD children, those with severe disability, children with ASD/GDD or ASD and children entering their first year of formal schooling to be seen earlier. This possibly reflected that the clinicians were likely prioritising children based on analysing this information available in the referral at the time of triage. Only less than one-tenth of our cohort was more than 7 years of age, and median age was 51 months highlighting that clinicians did prioritise older children around the age of school entry. There were no differences between waiting times for other vulnerable groups such as Indigenous status, OOHC, refugee and contact with DCJ. As the number of children with these vulnerabilities by our CDAS service was small, it is possible those children were prioritised and assessed for NDD by specific community paediatric clinics such as the child-at-risk, Indigenous and refugee clinics within our region.

The study demonstrated the variability of waiting times and inverse law of public health access to health services still operating in our region. The study also mapped the centres in our region that predominantly saw children (up to 90%) from the most disadvantaged regions needing more resources, whilst some areas with affluence continued to have shorter waiting times.

Although the centres were resourced with the same type of staffing with paediatricians and allied health, they all serviced their local populations which were a different demographic mix. The exact number of referrals received at each site was not collected in the CDAS dataset (as data was entered only after child was seen by the service), however it is highly likely that this number varied between sites. This may be the reason why the number of children seen were also different at each site (Facility C saw the greatest number of children), and therefore there were variations in wait times. Facility E had the shortest wait times despite second highest number of children seen due to more resources being shifted at this centre as it served greater disadvantaged populations. There is a need to integrate the number of referrals received at each site with our existing CDAS dataset to monitor and explain these variations further. The advantage that our CDAS re-design project offered was to develop a systematic and a centralised data collection of waiting times. This provided an administrative prospective data set for monitoring of wait lists.

Our work gains particular significance with the fact that NDDs are becoming more prevalent and there is an increasing pressure on the sustainability of public funded developmental services [3, 11, 16]. The diagnosis of ASD has increased by 270% since 2000 and in our study more than half of our population cohort were children with ASD [4]. There is widespread public and media concern about the length of waiting times, coupled with the variations between regions and vulnerability factors affecting access [11, 12, 14, 24]. Despite wait times being documented to be a major barrier to accessing timely services there were no systematic studies that the authors could find that have done analysis of waiting times and how it relates to diagnosis, level of delays and demographic variables [1, 14].

Though our waiting times are still not optimal, there is very little guidance on appropriate wait times for a CDAS service. Research in Child and Adolescent Psychiatry have demonstrated the ‘end-point’ of waiting for families as 7 months, after which families often lose hope and disengage with services [24]. Long waiting times are also associated with parental dissatisfaction of developmental services [12, 18].

Despite working with shuffling of staffing resources across centres, the study noted that the wait times did not reduce significantly between 2018 and 2021, however the service saw a significant increase in wait times in 2022 (median: 363 days, IQR: 269–420). This could possibly be a roll-over impact of the COVID-19 pandemic that affected face-to-face service access in 2020 and 2021. Many children who were reviewed on telehealth needed a follow-up face-to-face developmental assessment meaning that the same child had to be assessed twice. Similar increase in waiting times have been reported from services in the United Kingdom [25–27].

The re-design of our CDAS model of care occurred in 2016 as the service was unable to meet the growing needs across SWSLHD due to an upward trend in referral rates despite static staffing levels [21]. There was no systematic prioritisation or data collection mechanism to monitor waiting lists therefore a purpose-built central database was created with a priority allocation process to triage referrals. The study found that only 42.6% of children between 2018 and 2022 were seen within the priority allocated time or earlier. This is a target that needs additional resources and further streamlining of administrative processes.

The majority of children in the study cohort were boys (73.7%) in keeping with the evidence that male gender is associated with GDD and ASD [28]. Three-quarters of children seen by the service were 5 years or younger and children older than 5-years had shorter median wait times for their initial appointment. This could be because of a general agreement among clinicians in the service to defer prioritising very young children (2-years and under) for standardised tertiary developmental assessments. Instead these children are often advocated by the service through their referrers to expedite access to early intervention and education supports [16]. Some CDAS services have however focused on the preschool children to reduce their wait times [12].

Our developmental assessment service covers a large population with diverse levels of socio-economic demographics. The region has the highest intake of refugee population and many vulnerable cultural groups settle in this region [20]. The CALD sample formed 64% of our cohort and this is consistent with the population demographics of SWS [20]. Clinicians were aware of this factor and possibly looking out for CALD status information at triage resulting in shorter wait times for this group. Woolfenden et al. has proposed simplified referral pathways to early intervention to prevent children from CALD backgrounds “slipping through the net” [17].

Child development outcomes often follow a social gradient [16]. The study used IRSD deciles to determine particularly vulnerable regions. Almost 50% of patients seen by the service were living in suburbs classified as decile 1 or 2. Wait times fluctuated with IRSD deciles. This is crucial to monitor as there is a double challenge of social disadvantage and disability which likely still exists [16]. In addition, it is highlighted in research how personalised budgets for early intervention programs can potentially widen social and health inequities [29].

Our data analysis demonstrated that children with more severe GDD lived in disadvantaged suburbs and children with average cognitive skills lived in regions with socioeconomic advantage. It is known that Australian children with severe disability are more likely to live in low income households compared to high-income households (5.2% vs. 3.1%)^29^. The role of public funded developmental assessments thus becomes increasing vital as often sustained advocacy is needed to address the complex array of system and family level factors that influence access to early intervention [1].

### Implications for service delivery and future research

The implications for future service delivery include overall reduction in wait times, more transparent and consistent prioritisation of children that are assessed by the CDAS teams, linking with early intervention prior to assessment, and optimal utilisation of CDAS resources for assessment. The clinicians in the service have debated the CDAS Model of Care intensively based on research evidence. The model offers flexibility for meeting the needs of individual children and families based on psychosocial and medical complexities rather than a one-size-fits all approach. It was also noted that additional work is required in our CDAS re-design project to systematically document vulnerability factors as we could not demonstrate that we prioritised those children earlier.

There is a need for further discussion with service providers and studies on what service targets should be acceptable by CDAS teams. It was not possible from our study findings to make specific recommendations on what proportion of children should receive an appointment within the priority allocated category and the ideal wait time. There is some guidance on this to be 7 months based on prior research on wait times for access to child mental health services [24]. The study authors believe, as do previous researchers, that all children with complex neurodevelopmental concerns should have a comprehensive developmental assessment prior to school entry. Our study would also stimulate further research studies on the issue of wait times, and development of prioritisation processes by CDAS services.

An ideal CDAS system needs primary and secondary child health systems to be strengthened to ensure children start early intervention prior to a tertiary assessment. There is often a lack of coordination between different levels of systems for early identification, intervention, and referrals in both public and private health systems. There is a need to continue to work towards integration of various levels of health systems providing ambulatory services to children.

Our work highlights the need for multidisciplinary developmental clinics to maintain consistent data collection, monitoring and evaluation systems. We currently do not have systems to collect data on interventions that were accessed prior to the CDAS appointments. This would help monitor the changes seen in children with intervention, the impact of resources and which families are missing out on early intervention. Assessment of access to services according to IRSD provides a unique opportunity to target areas with high vulnerability. Service evaluation needs further qualitative research with parents to understand their experiences in accessing developmental assessments.

We also note that our prioritisation process is highly dependent on ‘clinician judgement’, and there is a need to develop a more consistent and transparent priority scoring system.

### Limitations

The limitations of this study include data entry errors and inconsistences related to multiple clinicians entering information into the database. This was mitigated as much as possible during the data cleaning and cross-checking processes. One important deficiency was the lack of data entry on the multidisciplinary level of allocation that we intended in our model of care along with prioritisation process. This would be most relevant to collect for future studies for transparent and consistent allocation of medical and allied health staffing for CDAS teams.

The project spans a four-year period, of which three years were during the COVID-19 pandemic, when clinical delivery systems were changed to telehealth. The database was not updated to record the modality of assessment (telehealth vs. face-to-face) due to competing priorities. This could possibly have an impact on waiting times and needs further exploration as there is potential to evolve CDAS model of care into a hybrid model of service delivery.

We also did not have data on other social-economic vulnerabilities at a family level such as parental education and psychosocial stressors. Instead, we used a population-based approach and SEIFA as a proxy of regional social disadvantage. We did not analyse data on children accessing other paediatric clinics that may have already provided developmental assessment within their service particularly for more vulnerable families such as out-of-home care, Indigenous and refugee families.

## Conclusion

This study was a retrospective analysis of data from the SWSLHD CDAS database following a service re-design. It provides the first evaluation of prioritisation, wait times, and the impact of suburban socioeconomic disadvantage at a population level on access to tertiary publicly funded child developmental assessments. In this health domain where demand is high, the study provides important insights into the profile of children seen and the challenges, complexity of service delivery and other factors affecting of wait times. The study could not demonstrate effectiveness of our prioritisation processes on wait times in different groups as more than half of children could not be seen within their allocated priority. The study however could demonstrate prioritisation of children from CALD backgrounds, severe disability, and ASD. There were no differences wait times for other vulnerable groups such as Indigenous, OOHC, and refugee backgrounds. Despite this, the study provides arguments for need of further research and discussion on benchmarking targets with regards to prioritisation and wait times. Further development of prioritisation system could include scoring systems that include factors such as ASD, severity of NDD, CALD status, and other vulnerability factors at the time of triage itself. There is also a further need to systematically collect and identify which children need more intensive multidisciplinary assessment compared to those that could benefit from paediatrician/allied health only assessments for effective resource allocation and utilisation. We envision that our research will further encourage researchers and clinicians to study wait times, prioritisation, resource allocation in other similar services.

### Electronic supplementary material

Below is the link to the electronic supplementary material.

Supplementary Material 1

Supplementary Material 2

## Data Availability

The de-identified datasets used and/or analysed during the current study have been made publicly available.

## Abbreviations

ABS: Australian Bureau of Statistics
ASD: Autism Spectrum Disorder
CALD: Culturally and Linguistically Diverse
CDAS: Child Development Assessment Service
DCJ: Department of Communities and Justice
GDD: Global Developmental Delay
IRSD: Index of Relative Socio-Economic Disadvantage
LGA: Local Government Area
LHD: Local Health Distract
NDD: Neurodevelopmental Disorders
OOHC: Out of Home Care
SEIFA: Socio-Economic Indexes for Areas
SWS: South Western Sydney
