# Supplementary material for: Access to child developmental assessment services in culturally and linguistically diverse metropolitan Sydney: a retrospective cohort analysis
Source: BMC Health Serv Res. 2024 Mar 14;24:342. doi: 10.1186/s12913-024-10800-y (PMC10941404; doi:10.1186/s12913-024-10800-y)
Supplement: Supplementary file 1 — Supplementary Material 1 [file 12913_2024_10800_MOESM1_ESM.docx]

**Child Development Assessment Service (CDAS)**

**Model of Care**

**South Western Sydney Local Health District**

# Overview

Developmental and diagnostic assessment is seen as the final / peak step of a longer screening and assessment process.

The proposed overall model of care for developmental and diagnostic assessment includes two separate models, one for pre-school children and one for school-aged children. All children progress through the same central intake and triage elements, after which the models diverge in order to better address individual and organisational needs.

The most significant changes from existing models of care include:

- Introduction of differing clinical pathways based on need, instead of a “one size fits all” approach.
- Specific triage, prioritisation and waiting list management.
- Consistent contact with families prior to the assessment, to ensure appropriate services and supports are being accessed.


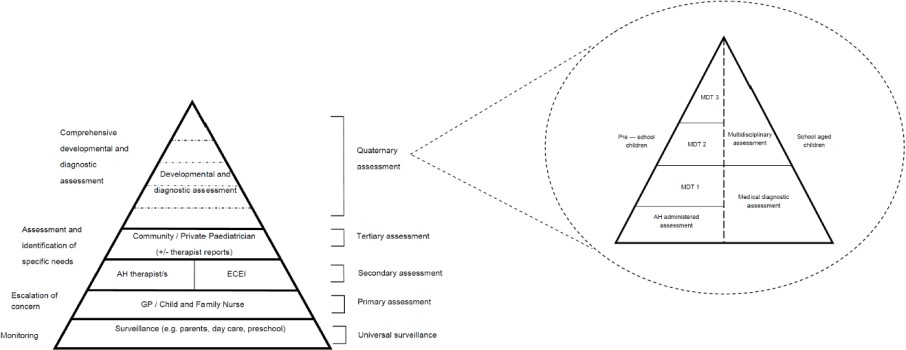


# Model Pathways

Pre-school pathway


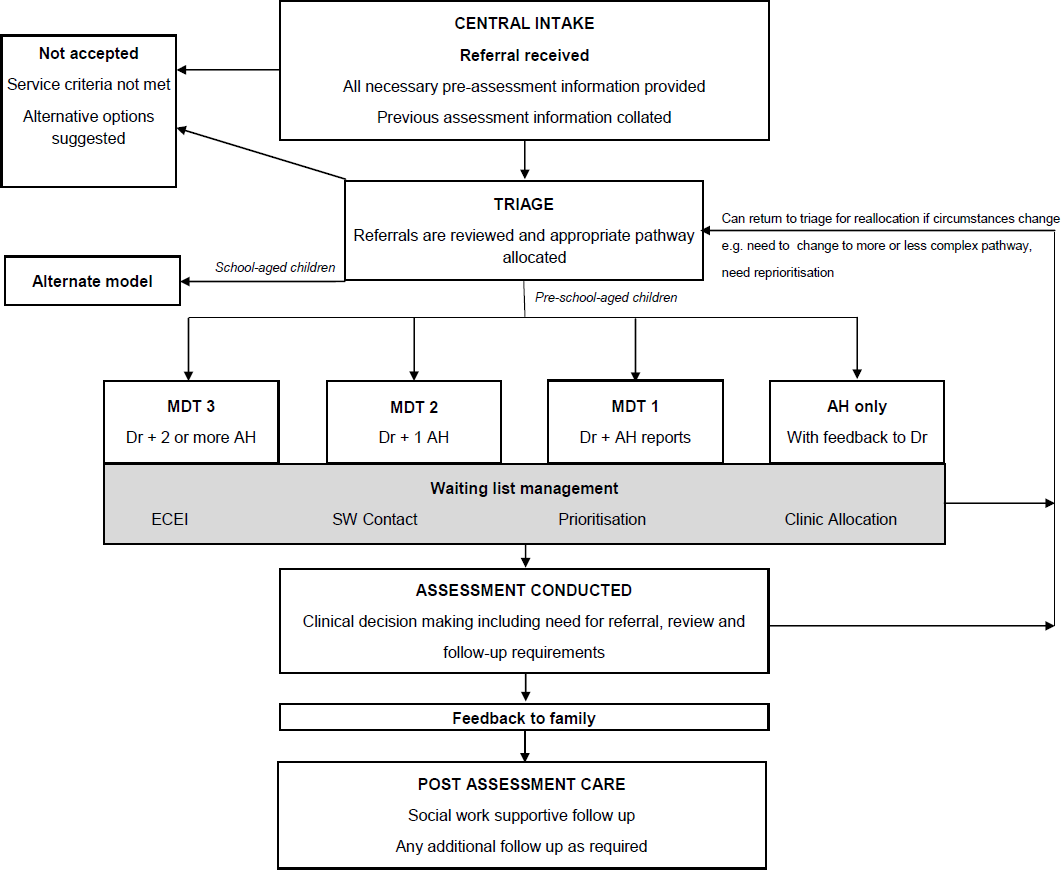


Criteria for pre-school pathway

| **MDT 3** | **MDT 2** | **MDT 1** | **AH Only** |
| --- | --- | --- | --- |
| Dr + 2 or more AH | Dr + 1 AH | Dr + AH reports (reports to be provided at intake) | Clinical Psychologist or other appropriately trained AH  + case discussion with Dr |
| - No previous AH assessment or therapy (from any AH discipline), particularly when psychosocial issues have resulted in families not seeking or accessing such services (e.g. family dysfunction, parental mental health issues, FACS involvement). - High diagnostic complexity – may have been through an AH only or MDT 1 pathway and diagnostic decision unable to be confidently applied. - ADOS needed for more thorough diagnostic assessment following initial contact through AH or MDT 1 pathways. (i.e. extremely complex diagnostic picture and unable to be resolved through AH or MDT 1 or 2 pathways) - Social worker present if very significant psychosocial concerns (e.g. severe family distress, child protection concerns) | - Children with autistic features reported (no diagnosis) and developmental assessment also required. - Some prior AH assessment and intervention, but potentially not all relevant disciplines (arrange for “missing” discipline to attend assessment). - Social worker attends with doctor if very significant psychosocial concerns (e.g. severe family distress, child protection concerns). - Reviews of progress following intervention/allowing for further development in order to fully clarify diagnosis and needs. - ADOS needed for more thorough diagnostic assessment following initial contact through AH or MDT 1 pathways. (i.e. highly complex diagnostic picture and unable to be resolved through AH or MDT 1   pathway). Not necessary to wait for an MDT 3 appointment. | - Has received prior AH assessment and intervention (reports provided or retrieved from eMR) appropriate to reported concerns (e.g. language – speech pathology, play/self-care /fine motor– OT, gross motor – PT) - May have medical concerns identified through intake and triage - May have been through AH only pathway and medical assessment required - Already have ASD diagnosis and developmental assessment needed and have concurrent medical concerns. - Have already had developmental/psycho metric assessment outside of this pathway (e.g. NICUS assessment, some children in PAHU, private) and medical evaluation required. | - Already have ASD diagnosis but developmental/psycho metric assessment required. - No associated medical concerns identified - Review developmental assessment (for children whose initial assessment was more than two years prior to transition to school) to review progress and support school transition. |

School-aged pathway


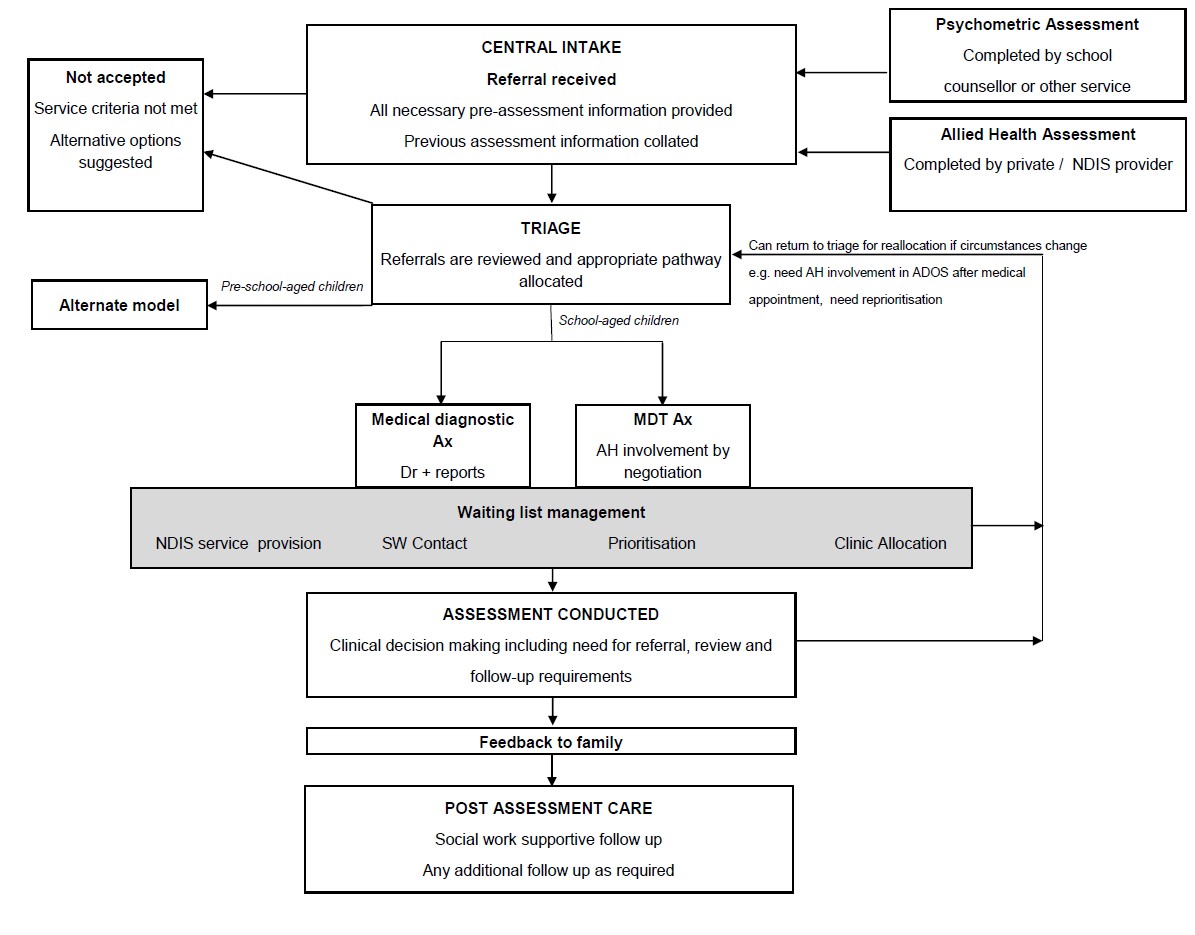


Criteria for school-aged pathway

| **Medical Diagnostic Assessment** | **Multidisciplinary Assessment** |
| --- | --- |
| Dr + AH & School Counsellor reports (reports to be provided at intake) | Dr + AH disciplines available by negotiation |
| - Majority of children will receive this pathway in first instance, and with the addition of a MDT Ax if diagnosis unable to be resolved. - (Note that SWSLHD allied health services do not routinely see school-aged children.) | - High diagnostic complexity – unable to resolve diagnosis through medical diagnostic assessment alone - ADOS needed for more thorough diagnostic assessment following initial contact through medical pathway. - Social worker present if very significant psychosocial concerns (e.g. severe family distress, child protection concerns) - Arrange appropriate AH disciplines for presenting concerns |

Notes/caveats:

- Referral information needs to be as comprehensive as possible to support effective triage into these pathways.
- Access to the pathways will depend on their availability. To prevent unnecessary waits, children triaged into a higher pathway may receive initial contact through a lower pathway if required.
- Role of registrars will vary depending on point of progress through training, current skill level and achievement of learning goals.
- Identifying key allied health staff to complete formal training on assessment tools i.e. Griffiths will help to establish and expand the pre-school AH pathway.

# Prioritisation Principles

These principles are used to guide prioritisation within pathways and ensure all clinicians work from the same perspective in prioritising. Independent clinical decision making is used to assign a priority where the significance of one factor outweighs another, or a client’s circumstances are unique.

1. Priority
   - Children who will attend school the following year will be prioritised for assessment before school entry.
   - Families with high psychosocial complexity/vulnerability (as flagged by the social worker)
   - Children who have not received any appropriate early intervention services (particularly if families have not engaged despite recommendations)
2. Next available
   - Children who do not have a diagnosis yet, but are accessing early intervention services
3. Safe to wait
   - Very new OOCH or refugee placement – wait until appropriately settled in new situation
   - Already have a diagnosis (e.g. ASD) and accessing NDIS and/or early intervention services
   - Very young children (2 years or younger)

Entry Criteria

Referrals are accepted from:

- Private and public paediatricians
- Paediatric neurologists
- Allied health from within NSW Health (including MAx screening clinics)

Eligibility:

Pre-school

Eligible

- Children with suspected developmental delay and/or ASD.
- Children aged 12 months to school entry
- Children should be referred to therapeutic services appropriate to their needs (e.g. Speech Pathology, Occupational Therapy etc) prior to their assessment.
  - Reports must be provided by families (if attending private services) or accessed through eMR (if accessing SWSLHD services) prior to triage.

Not Eligible

- Likely or suspected ADHD (without developmental delay) – redirect referral to a paediatrician experienced in ADHD management

School-aged

Eligible

- Children with suspected developmental delay and/or ASD
- Children who have commenced school, up to age 7.
- Children between 7 and 16 may be seen on a case-by-case basis. This may include children with complex/subtle symptoms, who have not been identified earlier. Consideration should also be given as to whether they are eligible to access other services, such as Headspace (eligible at age 12), and whether these may be more appropriate for adolescents who are in the process of transitioning to adult services.
- Children should be referred to therapeutic services appropriate to their needs (e.g. Speech Pathology, Occupational Therapy etc.) prior to their assessment through CDAT.
- Children should have received an assessment by their school counsellor and a language assessment prior to referral. Reports are to be provided prior to triage.

Not Eligible

- Likely or suspected ADHD (without developmental delay) – redirect referral to a paediatrician experienced in ADHD management
- “Pure” learning difficulties – redirect referral to school counsellor or private psychologist
- Require psychometric assessment only – redirect referral to school counsellor or private psychologist
- Children with mental health concerns other than ASD – redirect referral to ICAMS

Review Criteria

- Children are eligible for review assessment when:
  - There is diagnostic uncertainty, and the effect of intervention (e.g. therapy, preschool attendance) and further development needs to be determined before making a diagnosis / developing an appropriate longer-term intervention plan. These reviews typically occur within 6-12 months of the initial assessment.
  - Their school transition will occur more than 2 years after their initial assessment and diagnosis. Progress is reviewed and current needs are identified.
  - They are re-referred by their paediatrician as their current presentation is inconsistent with the diagnosis previously given.
- Social workers provide ongoing support until families are linked into appropriate services (including ECEI / NDIS)

Exit Criteria

- Children will be discharged from the service when:
  - Medical/MDT: assessment and reporting is complete, and they do not fit any of the review criteria.
  - Social work: when referred to/engaged with appropriate support services.

# Administrative management of referrals


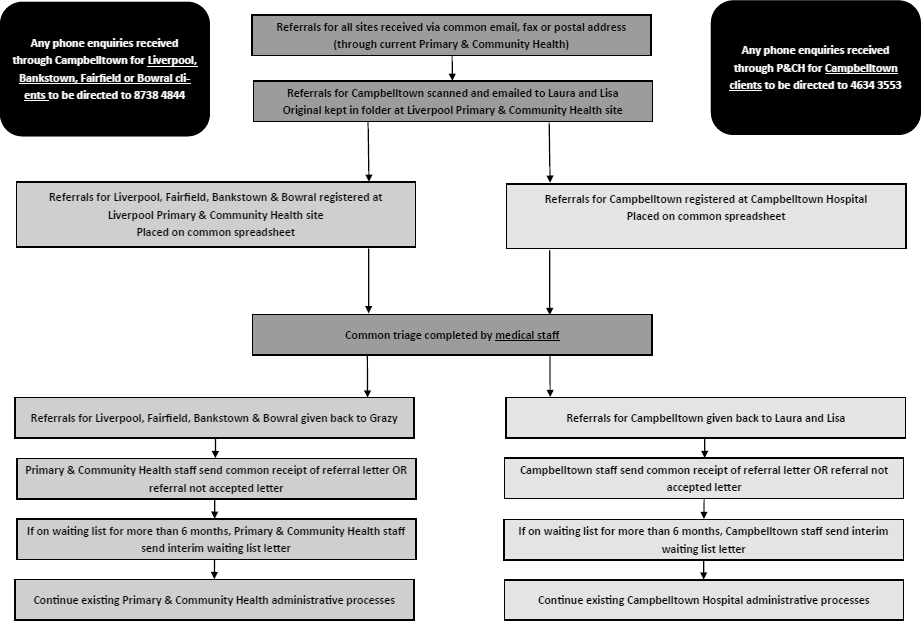


Referral and triage process

Information required prior to triage

- Referral via referral form or via letter covering all required criteria. Incomplete referrals will be returned to the referrer to provide further information.
- Reports or clinical documentation of interventions provided (family/service to provide if attending a private service, or team members to review eMR for SWSLHD services)
- Referrals may be returned for provision of further information if there is insufficient information provided initially

Triage meetings

A centralised triage meeting will operate for all district services.

Meetings are rostered fortnightly (however each person attends only once per month).

1. The first Monday of the month at 12:15pm for Fairfield, Bankstown and Bowral referrals.
   - Attended by Suky & Pankaj, and appropriate fellows/registrars
2. The third Thursday of the month at 2:30pm for Campbelltown and Liverpool referrals
   - Attended by Jo and/or Carolyn & Romy, and appropriate fellows/registrars Meetings are hosted from Liverpool with other sites teleconferencing in.

Documenting outcome of triage

- Outcome documented on eMR
- Enter child’s details, date of referral and planned date of assessment onto database
- Other local processes to continue
